# Influence and Mechanism of Solid-Phase Particle Factors on Oil–Solid Separation of Oily Sludge Treated by Flotation Method

**DOI:** 10.3390/toxics12120880

**Published:** 2024-12-02

**Authors:** Shuhui Wu, Xue Yao, Xiao Wang, Wenyan Yuan, Qiuhong Li, Xiaoyin Niu, Yanfei Ma

**Affiliations:** 1 School of Agricultural Engineering and Food Science, Shandong University of Technology, Zibo 255000, China; 15550340168@163.com (S.W.); yaoxue199901@163.com (X.Y.); 19811717922@163.com (X.W.); 2 School of Resource and Environmental Engineering, Shandong University of Technology, Zibo 255000, China; 17663731168@163.com (W.Y.); zbnxy@sdut.edu.cn (X.N.); 3 School of Materials Science and Engineering, Shandong University of Technology, Zibo 255000, China; qhli@sdut.edu.cn

**Keywords:** oily sludge, flotation, oil–solid separation, particle size, kinetics

## Abstract

The solid phase composition in oily sludge (OS) is a key factor affecting the oil–solid separation of OS. In this paper, the effects and mechanisms of solid-phase particle factors on the oil content of residue phase were investigated in order to improve the oil–solid separation efficiency. Flotation experiments were carried out on single-size sand and mixed-size sand OS consisting of three particle sizes at room temperature without adding flotation reagents. The effects of different-size particles as solid phase composition of OS and flotation parameter settings such as flotation temperature (Tp), flotation time (Tt), impeller speed (Rs) and liquid-solid ratio (L/OS) on the oil–solid separation efficiency were investigated. The experimental results showed that the oil content of residue phase decreased with the increasing of solid-phase particle size for single-size sand OS, and the optimal flotation conditions were Tp of 50 °C, Tt of 25 min, Rs of 1450 r/min and L/OS of 12:1. The oil–solid separation was more pronounced for mixed-size sand OS with a complex particle composition, while different particle compositions of the solid phase in OS promoted oil–solid separation. Scanning electron microscopy (SEM) and Fourier-transform infrared spectroscopy (FT-IR) characterisation of OS before and after flotation confirmed the relative advantage of coarse particle OS in the oil–solid separation process. The classical first-order model was well fitted to the flotation kinetic process of single-size sand and mixed-size sand OS. The response surface methodology (RSM) method was used to determine the Rs as the main control factor of the flotation process, and the oil content of residue phase in mixed-size sand OS was optimised to 2.63%. This study provides important process parameters and theoretical basis for the efficient treatment of OS.

## 1. Introduction

OS is the source of large and widespread pollution, generated in the petrochemical, steel-manufacturing and water treatment industries, and composed by a variety of substances, including oil, water, and solid particles. OS not only poses a serious threat to the environment, but also poses a major challenge to enterprises in terms of waste treatment and resource recovery. Properly and effectively treating OS can not only reduce environmental pollution, but also recycle useful resources from it. Therefore, conducting efficient research on OS treatment technology has important economic value and environmental significance. At present, the treatment technologies for OS mainly include thermochemical, mechanical, thermal treatment, and biological methods [1]. The thermochemical method can remove most of the light oil components but it is difficult to remove the heavy oil components effectively, and the chemicals may lead to the problem of secondary pollution [2]. Although the heat treatment method has high separation efficiency, its application scope is limited by its high energy consumption and high cost [3]. The biological method is environmentally friendly, but the treatment cycle is long and the treatment efficiency is limited [4]. The mechanical method is widely used for its low cost and simple equipment [5]. As a mechanical method, flotation has shown relative advantages in the treatment of OS due to its high efficiency, low cost and ease of operation.

There are numerous factors affecting the treatment of OS by flotation. In order to improve the efficiency of oil–solid separation, ref. [6] highlighted the application of nanobubble technology in oil sand mud flotation, which could reduce the size of nanobubbles and increase the number of nanobubbles to accelerate the recovery of asphaltenes through enhanced agitation, high dissolved gas content and surfactant concentration. Ref. [7] used a microemulsion system to treat OS in a flotation unit, and determined the optimal process conditions by modulating the microemulsion-to-petroleum-sludge ratio, temperature and time. Most researchers have improved the oil–solid separation efficiency of OS by process means, but there are fewer studies on the effect of the nature of oil–solid itself on the oil–solid separation effect. In recent years, more and more researchers have found that the particle size of solid phase particles has an important influence on the oil–solid separation effect when processing OS in different fields. Ref. [8] studied the removal of TPH from contaminated field soils and found that a single wash was more effective in removing TPH than washing after sieving, and the contact of particles of different diameters with each other promoted the separation of contaminants. Ref. [9] found in their study of hydrothermal treatment of OS that decreasing the solid particle size increased the oil–solid contact area and enhanced the adsorption of crude oil on the mineral surface, which led to difficulties in oil removal. Ref. [10] used three surfactants to assist in the sonication of OS, and, during the study, it was found that smaller particle sizes and excessive time resulted in the reorganisation of oil and particles, while particle size distribution also affects the ability of particles to accumulate in suspension. The oil droplets are usually encapsulated or adsorbed by solid particles in OS, and the size, shape, and surface properties of the particles may have an effect on oil–solid separation. The larger surface area of finer particles may increase the stability of the oil–water interface, and the finer particles have a lower collision efficiency so that the oil–solid separation efficiency is lower [11,12]. When OS oil–solid separation occurs, fine particle solids can fill in the pores between the oil droplets to form a site resistance effect, hindering the merging and settling of oil droplets. Fine solids can also be adsorbed on the surface of the oil droplets to form a protective layer, which improves the stability of the oil droplets and leads to emulsification. This stability can and may hinder the aggregation and uplift of oil droplets, thus reducing separation efficiency [13,14,15]. In addition, fine solids can alter the density and buoyancy of oil droplets, further affecting their flotation behaviour [16]. Therefore, it is important to study the influence of solid-phase particle size on the flotation effect to optimise the flotation process and improve the efficiency of oil–solid separation. The solids in the OS include primary minerals and secondary clay minerals, and most of the existing studies have been analysed by researchers from the perspective of simulating oil sands composed of sand, clay, bitumen and mixed water in a comprehensive manner, but the separate effects of primary minerals on oil–solid separation are not clear.

In this study, the effects and mechanisms of solid-phase primary mineral particle size on the treatment of OS by flotation were systematically analysed and investigated under room temperature and without the addition of other chemicals, while the process conditions were optimised. Firstly, the flotation factors of single-size sand OS with different particle sizes were systematically investigated by the flotation method. Secondly, the effects of particle heterogeneity and the inter-particle forces were investigated by flotation experiments using mixed-size sand OS. In particular, flotation selectivity evaluation was performed by comparing three flotation kinetic model fits. Finally, the flotation conditions for mixed-size sand OS were optimised using RSM to determine the main controlling factors of the flotation process. This study aims to provide theoretical guidance and practical basis for the application of the flotation method in industry, and further promote the application of flotation technology in the field of OS treatment.

## 2. Materials and Methods

### 2.1. Preparation of Materials

#### 2.1.1. Experimental Materials

Three different particle sizes of primary minerals (air-dried quartz sand: 110–160 mesh, 160–200 mesh and 200–300 mesh) were used as the solid phase composition. The crude oil was extracted from the Shengli Oilfield in Dongying, China, in which the proportions of saturated compounds, aromatics, resins and asphaltenes were 57.5%, 30.5%, 6.8% and 5.2%, respectively. Tap water was used as the flotation fluid in all experiments.

#### 2.1.2. OS Preparation

Laboratory-made OS was used in this experiment. Single-size sand OS was prepared by mixing uniform-particle-size quartz sand as solid phase with crude oil. Mixed-size sand OS was prepared using quartz sand with different particle size composition as solid phase. The crude oil was dissolved in toluene and mixed thoroughly with a sample of quartz sand, and the ratio of crude oil to quartz sand was 25% (wt/wt). Then, the obtained OS was heated continuously (60–70 °C) to ensure the complete evaporation of toluene, and it was stored in a fume hood for 7 d. The prepared OS samples were stored in a refrigerator at 5 °C before using.

### 2.2. Flotation Experiments

**Figure 1:**
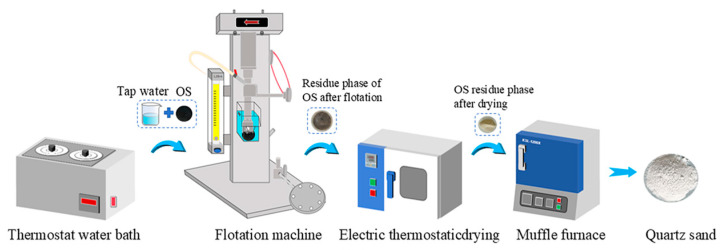
Schematic diagram of the experimental flow.

The flotation process was used to strip the oil phase from the surface of the solid phase by means of the shear force generated by the mechanical stirring of the impeller. At the same time, when air was injected into the flotation system, a large number of tiny bubbles was produced. The oil beads adhered to the bubbles and floated to form the oil phase, the middle layer was the water phase, and the sinking phase was the residue phase. Flotation experiments were carried out using a laboratory self-priming mechanical agitation flotation machine (HLXFD-0.5L, Wuhan, China). The temperature of the flotation solution was controlled by a thermostat water bath. In addition, 50 g of OS sample and flotation liquid were placed in the flotation tank at a certain liquid–solid ratio and stirred for 1 min to obtain the oil slurry. Then, the inlet pipe was opened, the airflow rate was 0.1 L/min, and the scraper continuously collected the upper oil phase at a rotational speed of 20 r/min. The flotation tank was regularly replenished with the flotation solution to maintain the total volume of the oil slurry at 600 mL. At the end of the flotation experiment, the flotation tank was removed and left to stand for 20 min. Then, the oil, water and residue phases were collected for further experimental analysis. The experimental flowchart of this study is shown in Figure 1.

#### 2.2.1. Single-Size Sand OS Flotation Experiment

The oil content of OS was kept at 25%, and three different particle sizes of quartz sand were mixed with crude oil to form a single-size sand OS: OS-A (110–160 mesh), OS-B (160–200 mesh) and OS-C (200–300 mesh). In the flotation experiments, the Tt was controlled at 10–35 min, the Tp was 30–70 °C, the L/OS was 3:1–15:1, and the Rs was varied from 1050 to 1650 r/min.

#### 2.2.2. Mixed-Size Sand OS Flotation Experiment

Keeping the oil content of OS at 25%, five samples of mixed-size sand OS100, OS80, OS60, OS50 and OS40 were prepared by changing the proportions of 200–300 mesh quartz sand to 100%, 80%, 60%, 50%, 40% of the solid phase. The remaining proportions of the solid phase were adjusted with the other two particle-size quartz sands, and the mixed ratio of 110–160 mesh and 160–200 mesh quartz sand was fixed at 1:1. The flotation experiments were controlled at Tt = 25 min, Tp = 50 °C, L/OS = 12:1 and Rs = 1450 r/min. At the end of the mixed-size sand OS flotation experiments, the water was dried, five groups of mixed-size sand OS residual phases were screened separately and calcined, and three granular residual phases (200–300 mesh, 160–200 mesh, and 110–160 mesh) were obtained initially and the oil content of residue phase was determined. In order to avoid aggregation of fine-grained solid phases into the coarse-grained range, a dispersant (sodium hydroxide, pH > 9.5–10) was added to the calcined residual solid phases, the three particle size volume fractions were determined and the preliminary oil content of residue phase was corrected. Finally, the oil content of residue phase was calculated separately for each particle size.

Each treatment of the above experiment was repeated three times and the data were expressed as the mean of three values.

#### 2.2.3. Optimisation Experiments for Oil–Solid Separation of Mixed-Size Sand OS

In this study, OS60 was used as the object of study. Rs (A), Tt (B), Tp (C) and L/OS (D) were selected as the four variables for optimisation using RSM with Box–Behnken design (BBD) [17,18]. The response value (Y) was the oil content of residue phase and a quadratic multiple regression equation was obtained and the experimental data were analysed using Design Expert 10.0 software.

#### 2.2.4. Flotation Kinetics Experiments

Flotation experiments were carried out on single-size sand and mixed-size sand OS, respectively, in accordance with the requirements of Section 2.2. Three Rs of 1050, 1450 and 1650 r/min were selected. The flotation intervals of 5, 10, 15, 20, 25, 30 and 35 min were used for calculating the cumulative oil recovery. Three flotation kinetic models were used to study the OS flotation performance, as shown in Table 1 [19,20,21,22]. The parameters of the models, such as maximum oil phase recovery (*ε*_∞_), flotation rate constant (*k*) and correlation coefficient (R^2^), were simulated with the help of analytical software (1stOpt 9.0).

#### 2.2.5. Analysis Methods

The samples collected at the end of the flotation experiments were used to determine the oil content of the residue phase by the differential gravimetric method. Firstly, the residue phase was put into an oven to dry the water at 105 °C for 4 h. To quantify the possible loss of the oil fraction at 105 °C, a thin layer of toluene-soluble oil was applied to the slide and dried at 105 °C for 4 h. The weight loss was measured to be within 2%, which is negligible. Then, it was transferred to a muffle furnace to calcine at 800 °C for 4 h to determine the solid content. Finally, the oil content of residue phase was calculated by the differential gravity method. The oil content of residue phase is defined as the weight of oil in the residue phase as a percentage of the total weight of oil solids.

Field emission SEM and Vector22 FT-IR (BRUKER, Karlsruhe, Germany) were used to observe the surface morphology and functional groups of the samples before and after flotation.

## 3. Results

### 3.1. Flotation of Single-Size Sand OS

#### 3.1.1. Effect of Flotation Temperature

**Figure 2:**
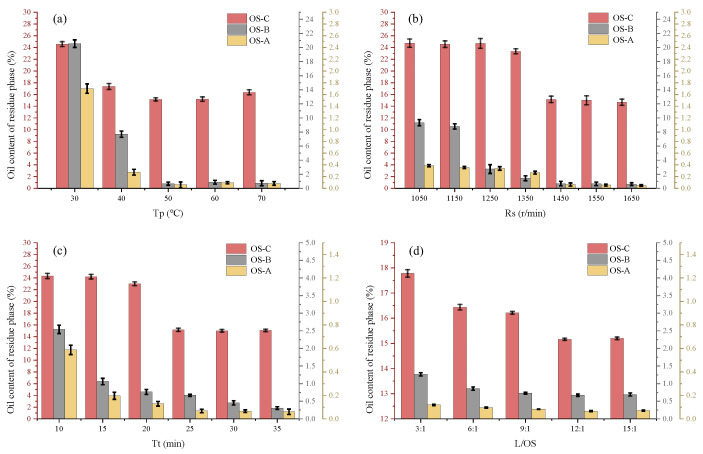
Impact of (**a**) Tp, (**b**) Rs, (**c**) Tt and (**d**) L/OS on the oil content of the residue phase of the single-size sand OS.

The impact of Tp (30, 40, 50, 60 and 70 °C) on the oil content of the residue phase in the single-size sand OS was investigated with the Rs of 1450 r/min, Tt of 25 min and L/OS of 12:1. The results are presented in Figure 2a. Figure 2a demonstrates that the oil content of residue phase in OS-A and OS-C decreased to 1.5% and 7.21% when the Tp was increased from 30 °C to 40 °C. As the Tp continued to increase to 50 °C, the decrease rate of the oil content of the residue phase in OS-A and OS-C slowed down. In contrast, the oil content of the residue phase in OS-B decreased continuously and significantly as the Tp increased from 30 °C to 50 °C. When the Tp was increased to 50 °C, the oil content of the residue phase in OS-A, OS-B and OS-C was 0.06%, 0.67% and 15.16%, respectively. The increase in Tp was favourable to reduce the oil content of residue phase in the single-size sand OS. The molecular activity and electrostatic repulsion between oil and solid phase increased with the increase in temperature, asnd the viscosity of OS and the adhesion between oil and solid phase decreased, which resulted in the oil phase being easily stripped [23,24]. The effect of Tp on the oil content of the residue phase in OS was related to the particle size of the solid phase in the OS. The finer the solid-phase particles of OS were, the less affected by Tp these were. This is due to the fact that a larger particle size (100–160 mesh) can provide more space and channels for oil phase, which promotes the movement and aggregation of oil droplets. Finer particle sizes (200–300 mesh) may reduce the mobility and separation efficiency of oil droplets due to close packing between particles. The oil content of residue phase changed insignificantly when the Tp was over 50 °C. It is worth noting that the high Tp was detrimental to oil–solid separation, and the change rule was consistent with the findings of [25]. This could be related to the emulsification of oil by mechanical agitation at higher temperatures, which significantly reduces oil viscosity. The reduced size of emulsified oil resulted in a lower flotation kinetics, thereby leading to more emulsified oil reporting to tailings [13,14]. Therefore, 50 °C was selected as the optimum Tp in the following experiments.

#### 3.1.2. Effect of Impeller Speed

The impact of Rs (1050, 1150, 1250, 1350, 1450, 1550 and 1650 r/min) on the oil content of the residue phase in the single-size sand OS was investigated for the Tp of 50 °C, Tt of 25 min and L/OS of 12:1. The results are shown in Figure 2b. From Figure 2b, it can be seen that the oil content of residue phase in OS-A, OS-B and OS-C decreased by 0.315, 8.66 and 9.57 percentage points with the increase in Rs from 1050 r/min to 1450 r/min, respectively. The increase in Rs had a greater effect on the oil content of residue phase for OS-B and OS-C. The oil content of the residue phase of OS-A at a lower Rs of 1050 r/min was 0.39%, and the oil–solid separation was so complete that increasing the Rs did not have a large effect on the oil content of the residue phase. On the one hand, as the Rs increases, both the fluid flow rate and shear rate increase, and turbulent fluctuations are intensified, thereby increasing the particle–bubble collision rate. On the other hand, the local pressure reduction induced by high-intensity agitation can increase the flow rate and overcome the attraction between water molecules, resulting in smaller-sized bubbles. The increase in the number of bubbles and the slowing down of the bubble rise rate increase the probability of collision with oil droplets, which enables the oil droplets to be transported upwards in time and prevents them from adhering to the solid phase again, thus reducing the oil content of the residue phase [26,27,28]. In addition, fine-particle OS with the same Rs was harder to be processed. Firstly, because the solid surface area of the fine particles is larger and the viscosity between the oil and the solid is higher. Secondly, the package pressure of the fine particles is enhanced at high Rs and they are more likely to be entrained into the upper oil phase mechanically [29,30,31]. Finally, the main factor in the shear separation of the oil–solid phase may be related to the thickness of the oil on the solid. When the oil phase content is certain, the smaller the solid-phase particles in OS, the larger the specific surface area, and the smaller the thickness of oil attached to the particle surface, which is not easily separated by shear. In addition, when the solid-phase particles in OS are larger, the proportion of oil in the inter-particle pores increases and is more easily removed by flotation. The oil content of residue phase in the three single-size sand OS was faintly reduced by increasing the Rs to 1650 r/min. Therefore, 1450 r/min was selected as the optimal Rs in the following experiments.

#### 3.1.3. Effect of Flotation Time

Figure 2c illustrates the impact of Tt (10, 15, 20, 25, 30, 35 min) on the oil content of residue phase in the single-size sand OS, under the conditions of Tp of 50 °C, Rs of 1450 r/min, and L/OS of 12:1. From the figure, it can be seen that the oil content of the residue phase in the three single-size sand OS showed a decreasing trend with the extension of the Tt. OS-A and OS-B were the first to reach the flotation equilibrium, and the oil content of the residue phase was 0.12% and 0.72% at 20 min, respectively. OS-C reached flotation equilibrium in 25 min, at which time the oil content of the residue phase was 15.16%. After reaching the flotation equilibrium and continuing to extend the Tt, the decreasing trend of the oil content of the residue phase in the three single-size sand OS was faint. This may be caused by the fact that the OS is highly homogeneously dispersed, and then most of the oil is successfully stripped by shear with the prolongation of the Tt. Further extension of the Tt did not significantly improve the oil–solid phase separation efficiency, which may be due to the limitation of the thickness of hydrodynamically stripped oil produced by shear at a certain Rs, which does not change with increasing Tt. The Tt in this study was set to 25 min based on the considerations of saving cost.

#### 3.1.4. Effect of L/OS

Figure 2d represents the impact of L/OS (3:1, 6:1, 9:1, 12:1, 15:1) on the oil content of residue phase of the single-size sand OS, with a Tp of 50 °C, Tt of 25 min and Rs of 1450 r/min. From the figure, it can be seen that the oil content of the residue phase of OS-A and OS-B decreased by 0.05 and 0.6 percentage points with the increase in L/OS from 3:1 to 12:1, which, in OS-C, was reduced by 2.62%. OS-C was more affected by L/OS compared with OS-A and OS-B. This is mainly due to the fact that a higher L/OS means that more water phase is involved, which contributes to better dispersion of the OS particles and makes the oil phase easier to be stripped by flotation, especially for OS-C containing finer-particle solid phase. When the L/OS value was increased to 12:1, the oil content of the residue phase tended to stabilise, and then, by increasing the ratio of L/OS, the oil content of residue phase increased. This may be due to the low concentration of solids, which reduces the efficiency of collision between particles and is detrimental to oil–solid separation, reducing flotation performance [32].

### 3.2. Flotation of Mixed-Size Sand OS

**Figure 3:**
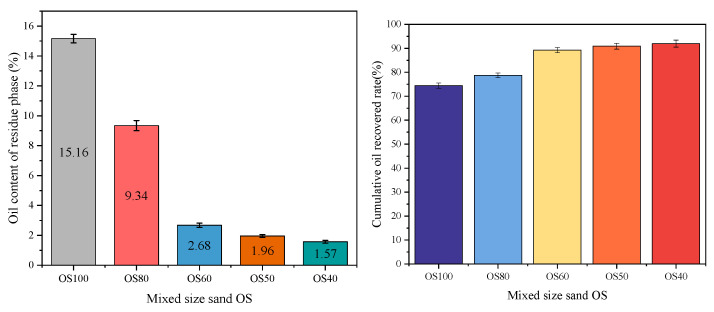
The oil content of the residue phase and cumulative oil recovery rate in mixed-size sand OS with different solid-phase particle configurations.

In order to further verify the effect of particle size of solid phase on the oil–solid separation efficiency in OS, as well as to explore the effect of particle-to-particle forces, mixed-size sands OS100, OS80, OS60, OS50 and OS40 were prepared by changing the proportions of 200–300 mesh quartz sand in solid phase for flotation experiments. The results are shown in Figure 3. The proportion of fine particles (200–300 mesh) was reduced from 100% to 60%, and the oil content of the residue phase from OS100 to OS60 decreased by 12.48 percentage points and the cumulative oil recovery rate increased by 14.8 percentage points. This may be related to the increased degree of heterogeneity of solid-phase particles in the mixed-size sand OS and the increased probability of inter-particle collisions. At the same time, the internal mobility of the OS was enhanced and the oil phase was more easily captured [33]. Continuing to reduce the percentage of fine particles in the solid phase, the oil content of residue phase was not significantly reduced.

**Figure 4:**
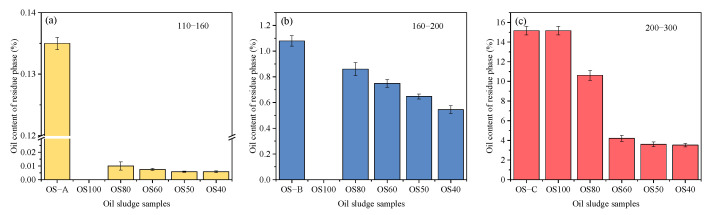
Compared oil content of the residue phases of (**a**) 110–160 mesh, (**b**) 160–200 mesh, and (**c**) 200–300 mesh after screening of mixed-size sand OS and oil content of the residue phase of single-size sand OS.

In addition, the oil content of the residue phase of three particle sizes obtained by separate screening of mixed-size sand OS is shown in Figure 4. From the figure, it can be seen that the oil content of the residue phase of all three sizes of solid phase particles after flotation screening of the mixed-size sand OS is reduced compared to that of the single-size sand OS. In the mixed-size sand OS obtained by mixing with fine particles, the initial oil thickness of the coarse solids in single-size sand and mixed-size sand OS samples was different due to the small specific surface area of the coarse particles and the decrease in the thickness of the surface oil, which may be a reason for the decrease in the oil content of the residue phase. From Figure 4c, it can be found that the oil content of the residue phase of OS80, OS80-300, OS50, and OS40 decreased by 4.62, 11.01, 11.6, and 11.68 percentage points, respectively, compared with that of OS-C (15.16%). Due to the larger specific surface area of fine-grained OS, the surface oil thickness was higher when mixed with coarse particles to formulate OS, and greater than that of OS-C. The result that the oil content of residual phases of fine particles was reduced after flotation in comparison with OS-C suggests that inter-particle interactions have a positive influence on the flotation effect of fine-particle OS, and that the mixing of coarse and fine sand improves the de-oiling of finer solids. Between solid phases, physical and chemical interactions may exist. Physical interactions are mainly through friction and collision between particles, which improves particle dispersion and stability as the percentage of coarse particles increases [34]. Regulating the particle size distribution directly affects the ability of particles to accumulate in suspension, which, in turn, affects shear force and apparent viscosity. As the proportion of solid-phase coarse particles in the OS increases, the shear stress increases and the apparent viscosity value decreases [35].

### 3.3. Flotation Kinetics of Single-Size Sand and Mixed-Size Sand OS

**Figure 5:**
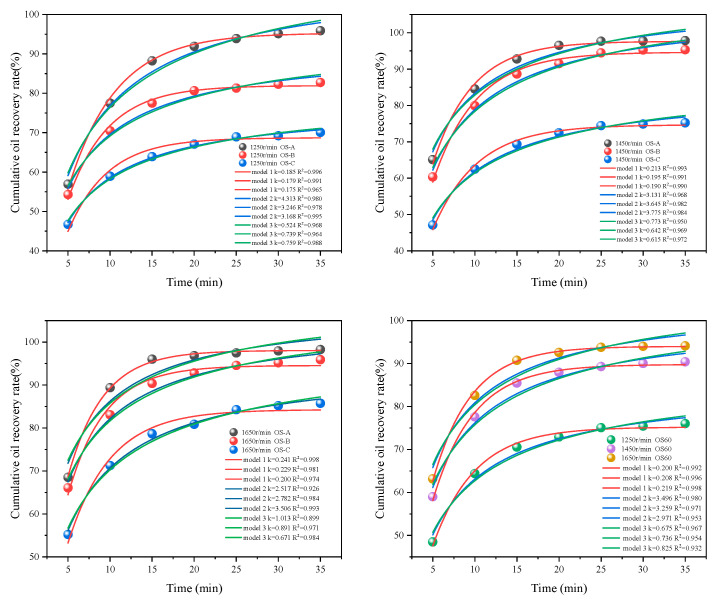
Kinetic fitting of different flotation models.

In order to further investigate the effects of particle size and Rs on the oil–solid separation effect, three different flotation kinetic models were selected and fitted to the cumulative oil recoveries of single-size sand OS and mixed-size sand OS60 at different Rs. The fitting results are shown in Figure 5 and Table 2. The graph displays a continuous upward trend in cumulative oil recovery for OS at a lower Rs of 1250 r/min with increasing Tt. When the Rs was increased to 1450 r/min, the increase in cumulative oil recovery from flotation for about 25 min OS was faint. However, under the Rs of 1650 r/min, the cumulative oil recovery rate of OS remained basically balanced at about 20 min. The time required for OS flotation to reach equilibrium decreased as the impeller speed increased. It was noted that the trend described above for OS-A and OS-B was observed, but there was a gradual upward trend for OS-C at three speeds. This was related to the previous conclusion that fine-grained sludge is more difficult to handle. At the same Rs, the OS with large solid-phase particle size had a faster oil phase recovery rate, higher cumulative oil recovery rate, and reached flotation equilibrium first. The data in Table 2 show that the R^2^ values of the first-order classical model are all greater than 0.9, which indicates that the model is fitted with high accuracy. It is also found that there is a maximum value of *ε*_∞_ exceeding 100% for model 2 and model 3, which may be due to the non-convergence of model 2 and model 3. In summary, the representative kinetics of the flotation process at Rs of 1250, 1450 and 1650 r/min are consistent with the classical first-order model [36]. Further analyses also revealed a positive effect of Rs and solid-phase particle size on flotation effectiveness. As the Rs becomes higher and the solid-phase particle size becomes larger, there is an increase in the k value (rate constant) of the model, leading to better selectivity of flotation [37].

### 3.4. SEM and FT-IR of OS and Residue

**Figure 6:**
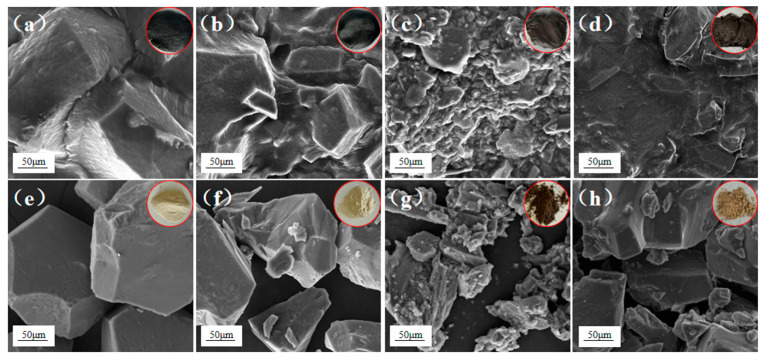
SEM and visualisation (top right corner) characterisation of OS from before and after flotation. (**a**,**e**) Single-size sand OS-A, (**b**,**f**) single-size sand OS-B, (**c**,**g**) single-size sand OS-C, (**d**,**h**) mixed-size sand OS60.

For further investigation of the mechanism of oil–solid separation, the morphology of the samples before and after flotation of single-size sand OS and mixed-size sand OS60 was characterised, and the results are shown in Figure 6. By comparing Figure 6a,e, it can be seen that the solid phase obtained from OS-A after flotation was almost pure quartz sand particles with an almost white colour. There were faint cohesive OS particles, no oil–phase interaction connection between particles, clear particle boundaries, and large pore sizes between particles. The comparison of Figure 6b,f shows that the solid phase obtained after OS-B flotation was pale yellow in colour, with a small amount of oil phase encapsulated on the surface of the particles, and the particles were still slightly adherent. According to Figure 6c,g, the colour of the solid phase obtained from OS-C after flotation was dark brown, and the particles were mostly adhered together into agglomerates without the presence of an obvious pore structure, and the flotation effect was poor. Before and after images of mixed-size sand OS60 flotation are presented in Figure 6d,h. The images show that most of the oil phase of OS60 was removed and the colour was brown. The surface of OS60 after flotation still had the oil phase encapsulated but the particles were not highly adherent with a relatively small inter-particle pore size and fewer pore structures. In summary, there was a significant difference in the morphology of OS, and the smaller the solid-phase particles in OS were, the larger the specific surface area was. Therefore, the higher the OS viscosity was, the tighter the inter-particle arrangement was; so, the pore connectivity was poorer. In addition, capillary interactions between fine particles and capillary pressure hindered crude oil flow and reduced the oil–solid separation efficiency [38,39,40,41].

**Figure 7:**
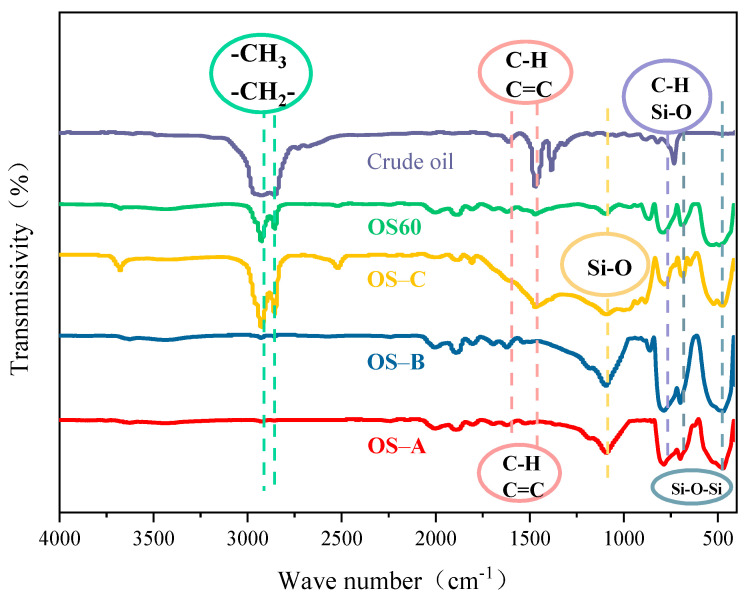
FT-IR spectra of crude oil and the residue phases of single-size sand OS and mixed-size sand OS60.

FT-IR was used to analyse crude oil and the residue phases of single-size sand OS and mixed-size sand OS60, and the results are shown in Figure 7. From Figure 7, it can be seen that absorption peaks appeared near the wavelengths of 2840–2960 cm^−1^, which were mainly caused by the C-H stretching vibration of methyl (-CH_3_) and methylene (-CH_2_-). These absorption peaks were usually associated with the presence of aliphatic hydrocarbons (such as alkanes), suggesting that the samples contained organic compounds with long-chain alkyls or methylene groups [42,43]. The peaks at 1620 cm^−1^ and 1470 cm^−1^ can be attributed to the C=C double-bond stretching vibration of the olefin or aromatic ring and the asymmetric stretching vibration of the methyl and methylene groups in the alkanes, respectively. The absorption peak at 1080 cm^−1^ may be caused by the stretching vibration of Si-O [44]. The peaks at 775 cm^−1^ and 680 cm^−1^ can be classified as symmetric bending vibrations of Si-O in quartz and out-of-plane bending vibrations of C-H in aromatic rings, respectively. The absorption peak at 509 cm^−1^ indicates the presence of the Si-O-Si [44]. The results of the FT-IR analyses showed a significant attenuation of the absorption peaks at 2840–2960 cm^−1^, 1620 cm^−1^ and 1470 cm^−1^ for the four OS after the flotation treatment, which proved the successful removal of most of the alkanes from the solid surface [45]. Meanwhile, comparing the intensity of the characteristic peaks, it was found that the oil in OS-A and OS-B was almost completely removed, and OS-C still contained a large amount of oil. Comparing the absorption peaks of OS60 and OS-C, it was found that the absorption peaks of OS-C were significantly stronger than those of OS60 at 2840–2960 cm^−1^, 1620 cm^−1^ and 1470 cm^−1^, which indicated that the oil removal effect of OS60 was superior than that of OS-C.

### 3.5. Optimisation of the Oil–Solid Separation Flotation Process by the RSM Method

In this study, RSM was used to investigate the interaction effect of response variables on the oil content of residue phase in mixed-size sand OS60 by considering four factors, A: Rs (1050–1650 r/min), B: Tp (30–70 °C), C: Tt (10–35 min), and D: L/OS (3:1–15:1), and the response value is the oil content of residue phase. The model was built for 29 experiments with four centre points. The experimental data including regression coefficients and analysis of variance (ANOVA) for the polynomial equations are shown in Table 3. Based on the obtained data, a quadratic multiple regression equation was obtained, as shown in the following equation.

Y = 5.78 − 9.13A − 0.99B − 2.58C − 1.27D + 0.59AB + 2.22AC + 1.75AD − 2.4BC + 0.09BD + 1.06CD + 5.69A^2^ + 1.21B^2^ + 0.59C^2^ − 0.95D^2^

The *p*-value of ABD under 95% confidence interval is less than 0.05, which indicates that the effect of this factor is significant, and the *p*-value of C is 0.1054, greater than 0.05, which is insignificant. In addition, the *p*-value of factor A is less than 0.0001, indicating that the effect of Rs on oil–solid separation is more significant than that of Tt, Tp and L/OS. The obtained *p*-values and f-values indicate that the regression model is statistically significant. The best predicted oil content of the residue phase was determined to be 2.63% under the optimal flotation conditions, which included an Rs of 1350 r/min, Tt of 25 min, Tp of 70 °C, and L/OS of 15:1. Validation experiments were carried out under this optimal flotation condition, and the oil content of residue phase was found to be 2.59%, which was only 0.04% different from the predicted result. It demonstrated the validity of the predicted results of the model [46]. Meanwhile, comparing the R^2^ value and the adjusted R^2^(Radj^2^), it can be found that the values of R^2^(0.9625) and Radj^2^(0.9250) were very close to each other. This result confirmed the adaptability and reliability of the model to the data, which indicated that the model was able to make good predictions [47].

**Figure 8:**
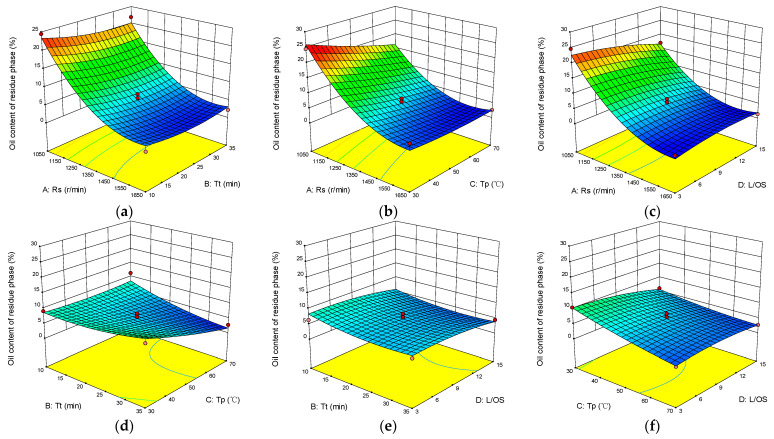
Response surface plots of the four-factor interaction of the oil content of residue phase: (**a**) the effect of Rs and Tt, (**b**) the effect of Rs and Tp, (**c**) the effect of Rs and L/OS, (**d**) the effect of Tt and Tp, (**e**) the effect of Tt and L/OS, and (**f**) the effect of Tp and L/OS.

The effect of the response variables and interactions on the oil content of the residue phase are shown in Figure 8. The results of Figure 8d,e show that the oil content of the residue phase decreases with the increase in the Tp and L/OS, and the effect of the Tt on the oil content of the residue phase is not significant. In addition, Figure 8a–c reveals that the oil content of the residue phase decreased significantly with the simultaneous increase in the response variables. The effect of Rs on the oil content of the residue phase was more pronounced than that of the other three factors. Moreover, it is worth noting that excessive Tp and L/OS resulted in higher oil content in the residue phase.

## 4. Conclusions

Solid-phase particles in OS have a significant impact on the treatment of OS by flotation for oil–solid separation. The conclusions of this study are summarised as follows. (1) The optimum flotation conditions were determined by single-size sand OS flotation experiments as follows: Tp of 50 °C, Tt of 25 min, Rs of 1450 r/min, L/OS of 12:1. Under these conditions, the oil content of the residue phase in OS-A, OS-B and OS-C reached 0.06%, 0.67% and 15.16%. (2) Through the mixed-size sand OS flotation experiments, it was found that the higher the degree of heterogeneity of solid-phase particles in OS, the better the OS flotation effect. There may be mutual reinforcement between the different particles. (3) The classical first-order model was well fitted to the flotation kinetic process of single-size sand and mixed-size sand OS. (4) The flotation experiments of RSM method for optimising oil–solid separation showed that the Rs had a more significant effect on the oil–solid separation of OS compared with the Tp, L/OS and Tt. The oil content of the residue phase of OS60 obtained was 2.59% under the optimal flotation conditions of Rs of 1350 r/min, Tt of 25 min, Tp of 70 °C, and L/OS of 15:1, which was only 0.04% different from the predicted result of 2.63%.

In summary, solid-phase particles have an important influence on OS oil–solid separation. Particle size of solid phase, particle heterogeneity and particle-to-particle forces play an important role in oil–solid separation. In this experiment, the effects and mechanisms of solid-phase particle factors on the efficient separation of oil–solid in OS treatment by flotation were investigated under room temperature and without adding flotation reagents. The treatment parameters were optimised to improve the oil–solid separation efficiency of flotation. In this way, the application of flotation technology in the field of OS treatment can be further promoted.

## Figures and Tables

**Table 1:** Three flotation kinetic models.

Number	Name	Formulas
Model 1	classical first-order model	$\varepsilon =\varepsilon_{\infty }[1-\exp(-kt)]$
Model 2	fully mixed factor model	$\varepsilon =\varepsilon_{\infty }[1-\frac{1}{\left(1+t/k\right)}]$
Model 3	second-order with rectangular distribution	$\varepsilon =\varepsilon_{\infty }\{1-[\frac{1}{\mathrm{kt}}\ln(1+kt)]\}$

*ε*: oil recovery at time *t*; *ε*_∞_: theoretical maximum oil recovery; *k*: flotation rate constant; *t*: time.

**Table 2:** Kinetic fitting parameters for different flotation models.

OS	Rs	Model 1			Model 2			Model 3		
		K	R^2^	*ε*_∞_	K	R^2^	*ε*_∞_	K	R^2^	*ε*_∞_
OS-A	1250 r/min	0.185	0.996	95.316	4.313	0.980	110.053	0.524	0.968	117.509
	1450 r/min	0.213	0.993	97.661	3.131	0.968	109.415	0.773	0.950	115.120
	1650 r/min	0.241	0.998	98.026	2.517	0.926	107.896	1.013	0.899	112.503
OS-B	1250 r/min	0.179	0.991	81.980	3.246	0.978	92.167	0.739	0.964	97.143
	1450 r/min	0.195	0.991	94.679	3.645	0.982	107.569	0.642	0.969	113.963
	1650 r/min	0.229	0.981	94.525	2.782	0.984	104.949	0.891	0.971	109.939
OS-C	1250 r/min	0.175	0.965	68.720	3.168	0.995	77.143	0.759	0.988	81.265
	1450 r/min	0.190	0.990	74.677	3.645	0.984	85.118	0.615	0.972	90.315
	1650 r/min	0.200	0.974	84.239	3.506	0.993	95.409	0.671	0.984	100.941
OS60	1250 r/min	0.200	0.992	75.180	3.496	0.980	85.105	0.675	0.967	89.998
	1450 r/min	0.208	0.996	89.769	3.259	0.971	100.952	0.736	0.954	106.407
	1650 r/min	0.219	0.998	93.986	2.971	0.953	104.829	0.825	0.932	110.039

**Table 3:** Analysis of variance (ANOVA) for the developed model.

Source	Sum of Squares	Df	Mean Square	F-Value	*p*-Value
Model	1412.00	14	100.86	25.65	<0.0001
A	1000.61	1	1000.61	254.51	<0.0001
B	11.78	1	11.78	3.00	0.1054
C	80.02	1	80.02	20.35	0.0005
D	19.37	1	19.37	4.93	0.0435
AB	1.37	1	1.37	0.35	0.5640
AC	19.65	1	19.65	5.00	0.0422
AD	12.28	1	12.28	3.12	0.0989
BC	23.24	1	23.24	5.91	0.0291
BD	0.029	1	0.029	7.452 × 10^−3^	0.9324
CD	4.49	1	4.49	1.14	0.3031
A^2^	209.85	1	209.85	53.38	<0.0001
B^2^	9.45	1	9.45	2.40	0.1433
C^2^	2.25	1	2.25	0.57	0.4617
D^2^	5.81	1	5.81	1.48	0.2442
Residue	55.04	14	3.93		
Lack of Fit	49.45	10	4.94	3.53	0.1175

## Data Availability

Data will be made available upon reasonable request.
